# Calculation of an Improved Stiffness Index Using Decomposed Radial Pulse and Digital Volume Pulse Signals

**DOI:** 10.3390/jpm12111768

**Published:** 2022-10-26

**Authors:** Hsien-Tsai Wu, Jian-Jung Chen

**Affiliations:** 1 Department of Electrical Engineering, Dong Hwa University, Hualien 97401, Taiwan; 2 Taichung Tzuchi Hospital, The Buddhist Tzuchi Medical Foundation, Taichung 42743, Taiwan; 3 School of Post-Baccalaureate Chinese Medicine, Tzu Chi University, Hualien 97002, Taiwan

**Keywords:** stiffness index (SI), ensemble empirical mode decomposition (EEMD), type 2 diabetes mellitus (T2DM), radial pulse, digital volume pulse, pulse wave velocity

## Abstract

The stiffness index (SI) is used to estimate cardiovascular risk in humans. In this study, we developed a refined SI for determining arterial stiffness based on the decomposed radial pulse and digital volume pulse (DVP) waveforms. In total, 40 mature asymptomatic subjects (20 male and 20 female, 42 to 76 years of age) and 40 subjects with type 2 diabetes mellitus (T2DM) (23 male and 17 female, 35 to 78 years of age) were enrolled in this study. We measured subjects’ radial pulse at the wrist and their DVP at the fingertip, and then implemented ensemble empirical mode decomposition (EEMD) to derive the orthogonal intrinsic mode functions (IMFs). An improved SI (SI_new_) was calculated by dividing the body height by the mean transit time between the first IMF5 peak and the IMF6 trough. Another traditional index, pulse wave velocity (PWV_finger_), was also included for comparison. For the PWV_finger_ index, the subjects with T2DM presented significantly higher SI_new_ values measured according to the radial pulse (SI_new_-RP) and DVP signals (SI_new_-DVP). Using a one-way analysis of variance, we found no statistically significant difference between SI_new_-RP and PWV_finger_ when applied to the same test subjects. Binary logistic regression analysis showed that a high SI_new_-RP value was the most significant risk factor for developing T2DM (SI_new_-RP odds ratio 3.17, 95% CI 1.53–6.57; SI_new_-DVP odds ratio 2.85, 95% CI 1.27–6.40). Our refined stiffness index could provide significant information regarding the decomposed radial pulse and digital volume pulse signals in assessments of arterial stiffness.

## 1. Introduction

Contour analysis of pulse waves is an important noninvasive method for assessing arterial stiffness based on photoplethysmography (PPG) pulses [1,2] or pressure pulses [3]. Recent studies have established that arterial stiffness increases with age [4] and is associated with cardiovascular risks, including hypertension [5] and the need for cardiac output monitoring [6], diabetes-associated arteriosclerosis [7,8], and end-stage renal disease [9]. Pulse waves, which provide an abundance of physical information, are signals comprising systolic and diastolic components. Systolic waves are forward-moving signals transmitted from the left ventricle to the location of measurement (e.g., finger or wrist), while diastolic waves flow backward from the lower limbs [2,3]. Among the typical methods for pulse wave contour analysis, determination of the stiffness index (SI) and related measurements represent easy and reliable approaches for determining the stiffness of large arteries in a clinical setting [2,3].

Millasseau and colleagues first proposed the method of dividing the height (in meters) by the time between the peaks of the systolic and diastolic components to obtain the SI parameter for use as an index [1]. In general, highly atherosclerotic subjects (e.g., older or diabetic) present diastolic components that flow backward faster than those of younger subjects (i.e., the time between the peaks of the systolic and diastolic components is shorter due to atherosclerosis) [1]. Although estimating the SI is easy for a pulse wave with prominent systolic and diastolic peaks, such as in young healthy subjects without significant arterial stiffness (i.e., with smaller SI values), the diastolic peaks are not well-defined (i.e., the diastolic component is not discernible) in older subjects and those with premature arterial stiffening, especially when assessed using photoplethysmography (PPG) [1]. Fortunately, several commercialized pulse wave analysis systems, such as the PulseTrace PCA2 (CareFusion, San Diego, CA, USA) [2,10,11] and PortaPres noninvasive hemodynamic monitoring device (FinaPres Medical Systems, Amsterdam, The Netherlands) [3], can accurately calculate the SI and related measurements even in very old patients or in those with cardiovascular disease. For example, researchers have measured the arterial stiffness index (ASI) at the index fingertip using finger photoplethysmography [2], finding that an increased ASI is associated with an increased risk of cardiovascular disease [2]. The authors of [3] defined the SI using the digital pulse propagation index (DPPI), based on the digital pressure pulse wave measured via a finger cuff. They found that a high DPPI was associated with established cardiovascular risk factors, including mean arterial blood pressure and smoking (all with *p* < 0.001).

In previous studies, we attempted to refine the diastolic pulse waveform with ensemble empirical mode decomposition (EEMD) [12] for SI computation [13,14,15] using prototype pulse analysis systems (i.e., an ECG-PWV system [13,14] and an air-pressure-sensing system [15]). EEMD is the first stage of the Hilbert–Huang transformation (HHT), which separates physiological signals into sets of distinct and orthogonal physiological information known as intrinsic mode functions (IMFs). After implementing EEMD, numerous orthogonal intrinsic mode functions (IMFs) are obtained. We initially chose IMF5 for SI computation, as it presented much larger amplitudes than the other functions, resembling the systolic and diastolic waveforms, and energy distribution analysis showed that its frequency distribution was between 1 and 4 Hz. We therefore selected IMF5 as the appropriate noise-free pulse wave [13,14,15]. We proposed that IMF5 most accurately represents the digital volume pulse (DVP) [13,14] and radial pulse [15] waveforms, and can be used as a basis for calculating the SI. However, our results were problematic. We had selected three subjects of different ages and health statuses included in [13,14] who presented distinct diastolic pulses according to the DVP measurements at the index fingertips. As expected, the younger adults exhibited lower SI values (subject A vs. subject C: 7.36 m/s vs. 10.30 m/s; Table S1). However, the SI values computed from IMF5 were higher for the younger adults (subject A vs. subject C: 8.44 m/s vs. 6.65 m/s; Table S1), which is illogical considering the definition of the SI. Some of the SI results based on IMF5 after EEMD among the four groups, measured according to the radial arterial waveforms at the wrist [15], are listed in Table S2 (young subjects vs. poorly controlled diabetes: 4.83 ± 0.63 vs. 2.77 ± 0.58 m/s). Unexpectedly, the poorly controlled diabetes group showed the smallest SI values, which is also illogical considering the definition of the SI. Thus, the second peak of IMF5 does not represent the diastolic pulse peak and cannot demonstrate the validity of the SI by itself.

Although the SI values derived from IMF5 alone showed statistically significant differences that could be used to differentiate between the groups in [13], the idea that IMF5 most accurately represents the DVP or pressure pulse waveforms through which the SI can be calculated is contentious. Therefore, there was an urgent need to develop an improved stiffness index, such as through using the decomposed radial pulse and DVP waveforms. We previously proposed interpretations of certain IMFs [15], as the interrelationships among the relevant IMFs are poorly understood, and could demonstrate the value of EEMD for signal analysis [16]. Our objectives are to determine: (1) an appropriate stiffness index for radial pulse and/or DVP signals after EEMD [17,18] and (2) a refined time-difference (ΔT_new_) parameter for the success of the new index (SI_new_). This manuscript is organized as follows: In Section 2, we define an appropriate stiffness index for the two decomposed pulse signals (radial arterial waveforms at the wrist and DVPs at the fingertip) after describing the study population, pulse signals and systems, and statistical analysis methods. In Section 3, we outline the refined time difference parameter for arterial stiffness assessment, measured between the first IMF5 peak and the IMF6 trough of the same heartbeat period, factors which were decomposed from DVP or RPP waveforms. Subsequently, we evaluate the performance of the new stiffness index by assessing its correlation with risk factors. To test the validity of the new index in translational signaling and medical applications, we compared the results with those obtained using the pulse wave velocity index by performing a one-way analysis of variance. We discuss the findings in Section 4 and conclude the manuscript in Section 5.

## 2. Materials and Methods

### 2.1. Study Population, Grouping, and Experimental Procedure

#### 2.1.1. Study Protocol

In this paper, we propose an improved stiffness index based on decomposed radial pulses and DVPs for arterial stiffness assessment in humans. Using the PWV index for comparison, we investigated variations in the radial pulse and DVP SI values of healthy middle-aged subjects and T2DM patients according to the differences in their clinical risk factors.

#### 2.1.2. Grouping

A. Inclusion criteria.

Eighty middle-aged participants (Group 1: *n* = 40, HbA1c < 6.5%; Group 2: *n* = 40, HbA1c ≥ 6.5%) were recruited for the final investigation (with de-identification of the data) between July 2009 and October 2011 in our previous studies [13,14,15]. All of the study subjects in Group 1 were recruited from a health-screening program at Hualien Hospital in Taiwan. The subjects in Group 2 attended a checkup every three months in the diabetes outpatient clinic at the same hospital (Table 1).

B. Exclusion criteria.

Subjects excluded from this study were those with a history of atherosclerosis-related complications, such as angina, myocardial infarction, stroke, and peripheral vascular diseases, within the past six months (as reported in our previous studies). We also excluded a minority of examinees who could not tolerate an upper-arm cuff pressure of up to 180 mmHg for 3 min during radial pulse signal measurement.

Table 1 lists the anthropometric and serum biochemical parameters of the two groups. The difference in age between the two groups was of little practical significance (*p* = 0.048 < 0.050).

#### 2.1.3. Experimental Procedure

All subjects fasted for 8 h before testing. On the day of measurement, the participants arrived at the outpatient clinic department in Hualien Hospital for medical assessment and blood sampling (including total cholesterol, triglycerides, low-density lipoprotein, high-density lipoprotein, fasting plasma glucose, and glycosylated hemoglobin). Subsequently, they waited to be assessed by a doctor outside the clinic. Following the medical assessment, the subjects arrived at a health clinic for physiological data recording (including age, body weight, height, waist circumference, and blood pressure), completion of the family-life questionnaire, and measurement of the radial pulse and DVP. We conducted all pulse measurements in a quiet room where the temperature was kept at 26 ± 1 °C. Firstly, we used the six-channel ECG-PWV system [7] for PWV_finger_ assessment and DVP signal measurement. The subjects rested for three minutes, and we then attached two pressure cuffs (i.e., a wrist cuff and an upper-arm cuff) in a refined air-pressure-sensing system (APSS) [15] to the left upper-arm and wrist for radial pulse measurement over 16 min. All the subjects provided written consent during the experimental procedure. The Institutional Review Board (IRB) of Hualien Hospital and Taichung Tzu Chi Hospital provided approval, and the data was used this study.

### 2.2. Measurement Instrument Description and Related Indices

#### 2.2.1. DVP and Radial Pulse Signal Measurements

PWV-ECG system for DVP measurement and PWV_finger_ index.

We measured the distance from points of reference in the sternal notch to the left-hand fingertip while the subjects were in the supine position (e.g., L_finger_). The six-channel ECG-PWV system [7] had a sampling frequency of 500 Hz, and we included the digitized signals acquired over 18 s (i.e., 9000 samples) for SI_new_-DVP offline computation. We also chose the R wave ECG at lead II as a reference point, and defined the time it took for a pulse to travel from this point to the left-hand fingertip as the time difference (T). Hence, the PWV_finger_ value was calculated as L_finger_/T, averaging the values over 18 s of successive cardiac cycles.

APSS for radial pulse measurement.

We included the original radial pulse signal measurements acquired at the wrist over representative 18-s periods during the first 5 min of wrist waveform collection (via the refined APSS [15] with a sampling frequency of 500 Hz) from the baseline phase only. The piezo-resistive sensor, which was connected to the second pressure cuff at the wrist, is widely adopted for the noninvasive measurement of blood pressure. We used this sensor for radial pulse wave detection and waveform analysis. The pressure detected by the piezo-resistive sensor was then converted into electrical signals prior to amplification and filtering to obtain the analog signals. After EEMD, we calculated the SI_new_-RP.

#### 2.2.2. SI Computation 

A. Computation of SI by DVP waveforms.

**Figure 1:**
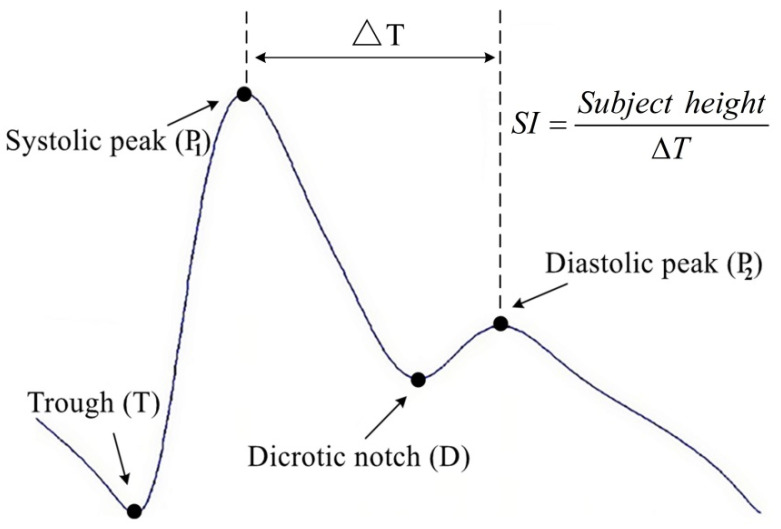
Measurement of digital volume pulse (DVP) by transmission of infrared rays through the finger. Generally, DVP comprises a systolic peak (the first peak) and a diastolic peak (the second peak). ΔT = the time difference between the first and second peak; stiffness index (SI) = body height/ΔT.

DVP waves are complex physical signals generally composed of systolic and diastolic components. The systolic components are the forward-moving waves transmitted from the left ventricle to the finger (the recording location), while the diastolic components are the backward-flowing signals produced when the pulse is transmitted along the aorta to the small arteries in the lower limbs. As shown in Figure 1, the stiffness index (SI) is a reliable index for determining the stiffness of large arteries [19,20]; a smaller value indicates a greater degree of stiffness. However, an important issue when using the SI to assess arterial stiffness is the ambiguity associated with the diastolic wave peak in older and atherosclerotic subjects (e.g., those with T2DM), which precludes accurate determination of the SI. Hence, many researchers have adopted EEMD to reconstruct the diastolic peak [21,22].

B. Ensemble empirical mode decomposition algorithm review.

This section starts with a brief review of the original empirical mode decomposition (EMD) method and ensemble EMD (EEMD). For a non-linear signal x(t), the EMD algorithm consisted of the following steps [18]:

(1) Connecting the sequential local maxima or local minima to derive the upper or lower envelopes using cubic spline, respectively.

(2) Averaging the upper and lower envelopes to derive the median of envelope, m(t).

(3) Extracting the temporary oscillation signal h(t) = x(t) − m(t).

(4) Repeat steps (1)–(3) on the temporary oscillation signal h(t) until m(t) is close to zero. Then, h(t) is considered as an IMF_n_(t).

(5) Compute the residue r(t) = x(t) − IMF_n_(t).

(6) Repeat steps from (1) to (5) using r(t) for x(t) to generate the next IMF (IMF_n+1_) and residue.

Subsequently, the EEMD algorithm first generates an ensemble of data sets obtained by adding different realizations of the white-noise signal w(t) to the input signal x(t). The EMD analysis is then applied to these new signal sets Y(t). Considering m and n as the number of realizations and the IMF index, respectively, the EEMD algorithm is then briefly summarized below in [17]:

(1) In each realization i, calculate the perturbed signal Y_i_(t):

(1) $$Y_{i}(t)=x\left(t\right)+ƞ\cdot \mathrm{std}[x\left(t\right)]\cdot w(t),$$

where ƞ is defined as the input noise amplitude, and *std* stands for the standard deviation operation.

(2) Apply EMD with N iterations to decompose Y_i_(t) into multiple IMFs.

(2) $$Y_{i}(t)=\sum_{n}^{}\mathrm{IMFi},n\left(t\right).$$

(3) Repeat step (1) and step (2) using a different series of white noise.

(4) The resultant IMF in EEMD is calculated as

(3) $${\mathrm{IMF}}_{n}(t)=\frac{1}{m}\sum_{k=1}^{m}\mathrm{IMFk},n\left(t\right).$$

In the current study, ƞ is set as 0.2, and N is equal to 200 for quick computing. Accordingly, EEMD is a useful nonlinear and nonstationary time-domain decomposition method. It is not only an adaptive, data-driven algorithm, but also decomposes a physiological signal (e.g., radial pulse or DVP) into limit number empirical modes in Equation (3), known as intrinsic mode functions (IMFs), orthogonal to each other [16,17,18]. Each orthogonal IMF represents a narrow band frequency–amplitude modulation that is often related to a specific physical process or some kind of noise.

C. An Appropriate Stiffness Index for Use after EEMD.

**Figure 2:**
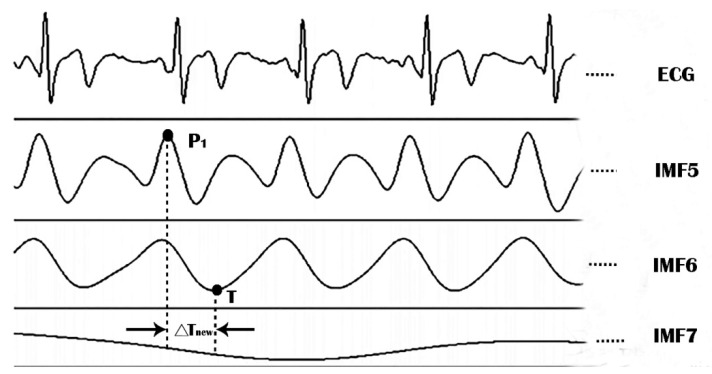
We chose IMF5 and IMF6 from Equation (3) to calculate SI indexes after decomposition of the radial pulse and DVP using EEMD. We automatically computed the time difference (ΔT_new_) between the first IMF5 peak (P_1_) and IMF6 trough (T) within the same period. We attributed IMF7 to respiratory signals, with a frequency of around 0.2 Hz. Since this signal did not affect the accuracy of the IMF5 signals, we deemed its measurement unnecessary.

In our previous studies [13,14,15], we chose IMF5 for SI computation, because it resembled systolic and diastolic waveforms, and energy distribution analysis showed that its frequency distribution was between 1 and 4 Hz. We therefore assumed that IMF5 was the ideal noise-free pulse wave. However, IMF6, generated by the impact of arterial pulsation on the pressure cuff or PPG sensor, exhibited a frequency close to that of the heart rate. The transit time (i.e., ΔT_new_) between IMF5 and IMF6 constitutes the time difference (in seconds) between the first IMF5 peak and the IMF6 trough within the same period (Figure 2).

Therefore, we defined the improved stiffness index (SI_new_) for use after EEMD as the ratio of body height to ΔT_new_ (Figure 2):

(4) $${\mathrm{SI}}_{\mathrm{new}}=\;\mathrm{body}\;\mathrm{height}\;/\;\Delta T_{\mathrm{new}},\;\mathrm{unit}:\;m/\sec.$$

To avoid confusion, we distinguished between SI_new_-DVP and SI_new_-RP as two different stiffness indices based on DVP and radial pulse signals, respectively.

### 2.3. Statistical Analysis

The values in Table 1 and Table 2 are represented as the means ± standard deviations (SDs) according to independent-samples *t*-tests. We assessed three arterial stiffness indices, namely, PWV_finger_, SI_new_-RP, and SI_new_-DVP. We analyzed the data using a one-way ANOVA followed by a Fisher’s least significant difference (LSD) post hoc multiple comparison analysis, and evaluated the association between SI_new_-RP and SI_new_-DVP using the Spearman correlation test. We also used this test, alongside the 95% confidence interval (95% CI), to measure the influence of the continuous parameters (e.g., anthropometric and serum biochemical parameters) in Statistical Package for the Social Sciences (SPSS, version 14 for Windows, SPSS Inc., Chicago, IL, USA). For the arterial stiffness indices calculation, we used the signal analysis software package in MATLAB R2020b (MathWorks Inc., trial use (30 days), USA). We developed a fitted binary logistic regression model based on the likelihood ratio estimates in SPSS to analyze the incidence risk of T2DM with different odds ratios.

## 3. Results

### 3.1. Use of EEMD for Decomposing IMF5 and IMF6 in Older and Diabetic Subjects 

**Figure 3:**
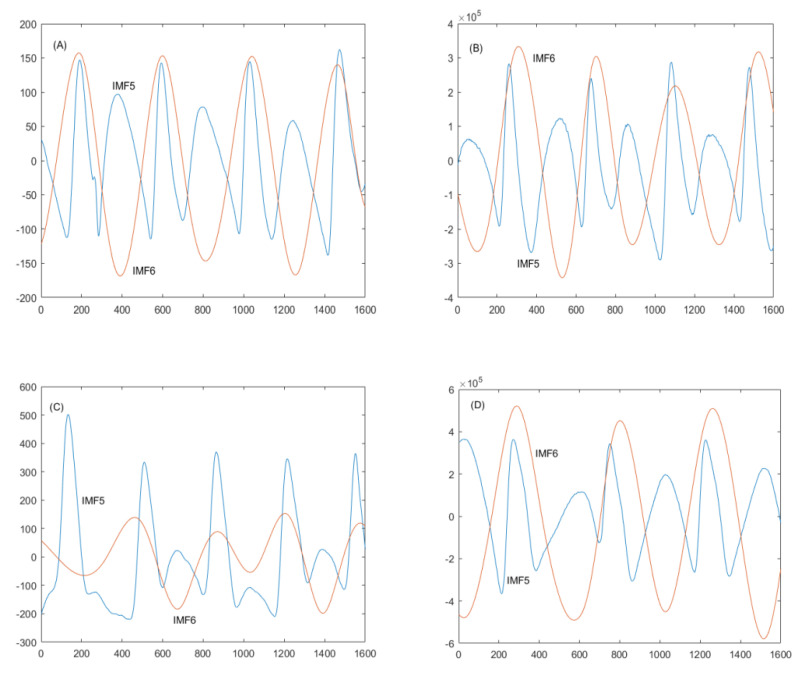
Variation in the radial pulse and DVP curve with age and diabetes. Non-stationary waveform signals were decomposed using ensemble empirical mode decomposition (EEMD) into nine intrinsic mode functions (IMFs), including IMF5 and IMF6. (**A**) Radial pulse from subject A (healthy older individual; 1.73 m, age 50) with ΔT_new_ = 0.43 sec and SI_new_-RP = 4.02 m/s; (**B**) DVP from subject A, with ΔT_new_ = 0.48 sec and SI_new_-DVP = 3.60 m/s; (**C**) radial pulse from subject B (type 2 diabetic patient; 1.63m, age 52) with ΔT_new_ = 0.28 sec and SI_new_-RP = 5.82 m/s; (**D**) DVP from subject B, with ΔT_new_ = 0.38 sec and SI_new_-DVP = 4.29 m/s. The graphs show lower ΔT_new_ values for the type 2 diabetic patient regardless of the signal type (radial pulse or DVP). For ease of viewing, the radial pulse and DVP signals of only 1600 samples are shown.

Figure 3 shows the effectiveness of decomposing IMF5 and IMF6 for assessing arterial stiffness (i.e., SI_new_-RP and SI_new_-DVP) in a middle-aged diabetic patient, as reflected by the higher value compared with that obtained for the middle-aged non-diabetic subject (5.82 m/s vs. 4.02 m/s for SI_new_-RP; 4.29 m/s vs. 3.60 m/s for SI_new_-DVP, respectively). EEMD [17,18] was applied to radial pulse and DVP signals using Equation (3) for decomposition of IMF5 and IMF6 to calculate the SI_new_-RP and SI_new_-DVP indices via Equation (4). The resulting values allowed distinguishing between the diabetics (Group 2) and non-diabetics (Group 1) in the population of similarly aged subjects based on statistically significant differences (all *p* < 0.05) (Table 2).

### 3.2. One-Way ANOVA for Three Arterial Stiffness Indices 

We compared the mean performance of the three arterial stiffness assessment indices (PWV_finger_, SI_new_-RP, and SI_new_-DVP) using one-way analysis of variance at a 0.05 level of significance, and the results are presented in Table 3(A). The mean performance of PWV_finger_ and SI_new_-RP were similar (4.90 vs. 4.96 m/s, respectively). The *p*-value for source of variance indicated significant difference between indices (*p* < 0.001); therefore, we applied Fisher’s least significant difference (LSD) post hoc multiple comparisons analysis, as presented in Table 3(B). The results of this analysis revealed that the mean score obtained for PWV_finger_ was similar to that of SI_new_-RP (mean difference = −0.058; *p* = 0.658) but not to that of SI_new_-DVP (mean difference = 0.524; *p* < 0.001). Finally, the results revealed that the mean score of SI_new_-RP, which presented a high level of stiffness, differed significantly in comparison to that of SI_new_-DVP (mean difference = 0.582; *p* < 0.001), which presented a low level of stiffness.

In other words, we found no statistically significant differences in the arterial stiffness assessment based on PWV_finger_ and SI_new_-RP for the same test subjects.

### 3.3. Correlation between SI_new_-DVP and SI_new_-RP

**Figure 4:**
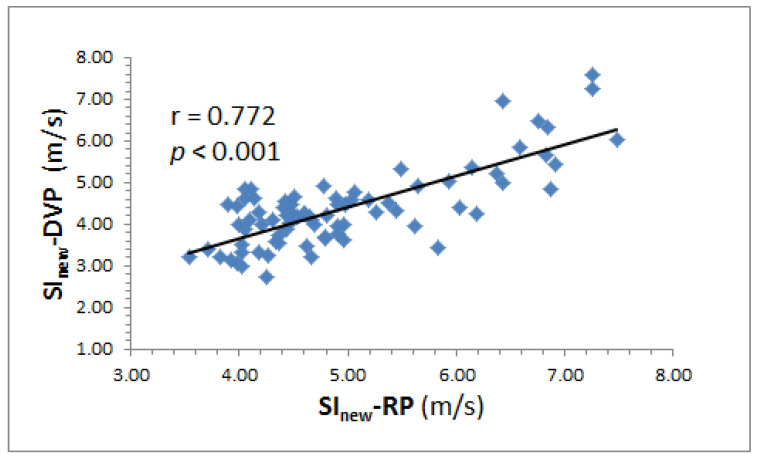
Significant correlation between the SI measured by six-channel ECG-PWV system [7] and SI calculated using the new method based on decomposed radial pulse signals (measured by a refined air pressure sensing system) [15]. SI_new_-RP—SI calculated using the new method based on decomposed radial pulse signals proposed in Equation (4); SI_new_-DVP—SI calculated using the new method based on decomposed DVP signals proposed in Equation (4).

We found no statistically significant differences between the two indices SI_new_-RP and PWV_finger_ (4.96 ± 0.98 vs. 4.90 ± 0.42 m/s, *p* = 0.658; mean difference 0.058 ± 0.218 m/s) (Table 3). Accordingly, SI_new_-DVP was significantly correlated with SI_new_-RP (r = 0.772, *p* < 0.001) (Figure 4). Both SI_new_-RP and SI_new_-DVP were significantly correlated with age, body weight, waist circumference, glycosylated hemoglobin, and triglyceride (Table 4).

### 3.4. Effects of Risk Factors on SI_new_-RP and SI_new_-DVP Indices

Among the 80 test subjects, 40 were healthy and 40 had T2DM. Subjects with T2DM had significantly higher arterial stiffness than those without T2DM, as determined by either radial pulse (4.51 ± 0.49 vs. 5.41 ± 1.13 m/s, *p* < 0.001, 95% CI 0.38 to 1.42 m/s) or DVP (4.02 ± 0.61vs 4.74 ± 1.09 m/s, *p* < 0.001, 95% CI 0.56 to 1.26 m/s).

Binary logistic regression analysis showed that subjects with t2dm had higher si_new_-rp and si_new_-dvp scores than those without t2dm, as determined by both novel arterial stiffness indices (si_new_-rp odds ratio 3.17, 95% ci 1.53 to 6.57; si_new_-dvp odds ratio 2.85, 95% ci 1.27 to 6.40; hosmer–lemeshow test *p* = 0.336 vs. *p* = 0.241, respectively).

## 4. Discussion

Commercialized pulse wave analysis systems, such as PulseTrace PCA2 [2,10,11] and the PortaPres noninvasive hemodynamic monitoring device [3], can accurately determine SI values, even for very old subjects or those with cardiovascular disease. However, the DVPs of most type 2 diabetic patients and the radial pulses of all subjects as measured by our prototype systems [13,14,15] did not present diastolic peaks and therefore preclude the direct computation of the stiffness index. In our earlier work, we demonstrated that EEMD could be applied rapidly and easily for computation of the stiffness index based on IMF5 [13,14,15]. Although this system was found to differentiate between the groups based on statistically significant differences, the fact that the SI values were lower in the older and type 2 diabetic patients was problematic. In the current study, we developed a new method for calculating the stiffness index using radial pulse and DVP signals subjected to EEMD (Figure 3) that demonstrated high efficacy for measuring arterial stiffness (Table 2). As with the PWV index, a higher SI_new_ value was associated with the presence of risk factors (Table 4). In our proposed method, we improve the accuracy of calculating the stiffness index (SI_new_) by adjusting the time difference according to the distance between the first IMF5 peak and the IMF6 trough within the same period (ΔT_new_) for subsequent EEMD (Figure 2). Our findings indicate that the method for calculating SI_new_ based on DVP and radial pulse signals is feasible for assessing arterial stiffness, though determining the feasibility for clinical application requires further evaluation.

Many studies have indicated that the wrist pressure pulse is related to the DVP [23]. Whereas SI_new_-DVP uses measurements from the fingertip, SI_new_-RP relies on air-pressure pulse signals from the wrist. The calculated SI_new_-RP index values corresponding to high stiffness differ significantly from the SI_new_-DVP index values corresponding to low stiffness (mean difference = 0.582; *p* < 0.001) (Table 3); Group 1 vs. Group 2 = 4.51 ± 0.49 vs. 5.41 ± 1.13 m/s for SI_new_-RP and 4.02 ± 0.61 vs. 4.74 ± 1.09 m/s for SI_new_-DVP, respectively (all *p* < 0.05, Table 2). The arterial stiffness assessments of the SI_new_-DVP and SI_new_-RP indices presented statistically significant differences (*p* < 0.001) for the same test subjects. Conversely, SI_new_-DVP was significantly correlated with SI_new_-RP (r = 0.772, *p* < 0.001) (Figure 4). Thus, the SI_new_ values provided by the two techniques (SI_new_-DVP and SI_new_-RP) are not identical. Determination of the SI_new_-RP index (i.e., pressure sensing) is more easily implementable on a wearable bracelet [24], whereas the DVP, unlike the stable radial pulse, is suitable for innovative wearable technology applications due to its advantages as a noninvasive and convenient diagnostic tool [23]. For example, PPG sensors could be used in eardrops, finger cots, and toe sleeves for acquisition of peripheral volume pulse information from the ear, index finger, and toe, respectively.

Arterial stiffness is an independent prognostic indicator of cardiovascular risk, and arterial stiffness is typically measured according to the pulse wave velocity [25,26,27]. The CAVI is also an important and independent risk marker in patients with chronic and acute coronary heart disease [28,29]. Values determined for our prototype index (SI_new_-RP) were not significantly different, statistically, from the PWV_finger_ index calculated for the same test subjects (mean: 4.90 vs. 4.96 m/s; mean difference: −0.058; *p* = 0.658) (Table 3(B)). However, binary logistic regression analysis showed that subjects with T2DM presented higher SI_new_-RP and SI_new_-DVP values than those without T2DM (SI_new_-RP odds ratio 3.17, 95% CI 1.53 to 6.57; SI_new_-DVP odds ratio 2.85, 95% CI 1.27 to 6.40; Hosmer–Lemeshow test *p* = 0.336 vs. *p* = 0.241, respectively). Therefore, our findings for wrist air-pressure pulse measurement were consistent with those in [23,24,30], which indicates the efficacy of using radial pulse waves in many applications. Finally, the optimized EEMD algorithm used in the current study can operate in real-time and at a sampling rate of up to 3500 Hz [17]. Therefore, our EEMD algorithm for decomposing IMF5 and IMF6 for SI_new_-RP and SI_new_-DVP calculation is a computationally efficient method that can be applied to large datasets in clinical applications.

This study has some limitations. Both groups comprised middle-aged subjects. Hence, this was not a wide-ranging clinical study, and the number of participants was limited. Furthermore, the subjects were not age-controlled (*p* = 0.048 < 0.050) to allow for unbiased analysis. This was an outpatient clinic-based cross-sectional study that relied on the accuracy of the data reported by the subjects. Therefore, a gap existed between the middle-aged asymptomatic group and the diabetic group. Although a positive association exists between heart rate and T2DM risk, average heart rate was significantly different between in the two groups (about 75 vs. 88 bpm) in our study, so it was difficult to prevent bias when grouping. We acquired 9000 wrist radial pulse and 9000 DVP data samples from our own dataset for computation of the SI_new_-RP and SI_new_-DVP under the same standard processes. As a result, the details regarding diet, exercise, and the medical control of diabetes may be incomplete and, thus, errors are inevitable. Finally, we recommend that future studies be conducted to include comparisons with other commercialized pulse wave analysis systems.

## 5. Conclusions

This study successfully solved the controversial problem of decomposing the radial pulse or DVP without discernible diastolic peaks to calculate stiffness index. Our results demonstrated that using a refined time difference between the first peak of IMF5 and the trough of IMF6 (i.e., ΔT_new_) for the decomposed signals with EEMD may serve as a useful tool, not only in the early detection of cardiovascular disease, but also in assessing disease progression in the area of translational signaling and medicine.

## Figures and Tables

**Table 1:** Anthropometric and serum biochemical parameters of Group 1 (healthy middle-aged subjects) and Group 2 (type 2 diabetic subjects (T2DM)).

Parameter	Group 1	Group 2	*p*-Values
Gender (male/female)	40 (20/20)	40 (23/17)	N/A
Age (years)	52.45 ± 10.99	57.80 ± 7.45 *	0.048
Body height (cm)	163.84 ± 7.83	163.83 ± 7.39	0.994
Body weight (kg)	64.08 ± 11.72	73.96 ± 11.43 **	<0.001
WC (cm)	82.58 ± 11.43	93.90 ± 10.17 **	<0.001
BMI (kg/m^2^)	23.79 ± 3.69	27.54 ± 3.89 **	<0.001
SBP (mmHg)	121.20 ± 14.36	121.65 ± 27.91	0.928
DBP (mmHg)	75.63 ± 8.57	75.30 ± 17.16	0.915
PP (mmHg)	45.58 ± 11.92	46.83 ± 15.81	0.691
HR (beats/min)	75.19 ± 17.22	87.65 ± 14.81*	0.001
HDL (mg/dL)	53.18 ± 17.21	40.53 ± 14.97*	0.001
LDL (mg/dL)	120.15 ± 41.09	128.45 ± 34.29	0.330
Cholesterol (mg/dL)	175.60 ± 50.63	190.83 ± 46.55	0.165
Triglyceride (mg/dL)	97.30 ± 47.32	171.23 ± 85.22 **	<0.001
HbA1c (%)	5.77 ± 0.34	8.86 ± 2.11 **	<0.001
FPG (mg/dL)	96.85 ± 16.08	169.08 ± 48.91 **	<0.001

The total number of test subjects was 80. Group 1, middle-aged asymptomatic subjects; Group 2, middle-aged type 2 diabetic subjects. WC, waist circumference; BMI, body mass index; SBP, systolic blood pressure; DBP, diastolic blood pressure; PP, pulse pressure; HR, heart rate; HDL, high-density lipoprotein cholesterol; LDL, low-density lipoprotein cholesterol; HbA1c, glycosylated hemoglobin; FPG, fasting plasma glucose. ** *p* < 0.001, * *p* < 0.05, Group 1 vs. Group 2. *p* values larger than 0.05 indicate differences are not statistically significant.

**Table 2:** Stiffness indices for large arteries (PWV_finger_, SI_new_-RP, and SI_new_-DVP) in Group 1 (healthy middle-aged subjects) and Group 2 (type 2 diabetic subjects).

Parameter	Group 1	Group 2	*p*-Values
PWVfinger (m/s)	4.76 ± 0.45	5.04 ± 0.34 *	0.002
SInew-RP (m/s)	4.51 ± 0.49	5.41 ± 1.13 **	<0.001
SInew-DVP (m/s)	4.02 ± 0.61	4.74 ± 1.09 *	0.001

The total number of test subjects was 80. Group 1—middle-aged asymptomatic subjects; Group 2—middle-aged type 2 diabetic patients. PWV_finger_—pulse wave velocity, measured by the PWV-ECG system [7]; SI_new_-RP—SI calculated using the new method based on decomposed radial pulse signals proposed in Equation (4); SI_new_-DVP—SI calculated using the new method based on decomposed DVP signals proposed in Equation (4). ** *p* < 0.001, * *p* < 0.05, Group 1 vs. Group 2. *p* values lower than 0.05 indicate differences are statistically significant.

**Table 3:** (**A**). One-way analysis of variance for the three indices (PWV_finger_, SI_new_-RP, and SI_new_-DVP) applied to Group 1 (healthy middle-aged subjects) and Group 2 (type 2 diabetic subjects). (**B**). Fisher’s LSD post hoc test for the three indices (PWV_finger_, SI_new_-RP, and SI_new_-DVP) applied to Group 1 (healthy middle-aged subjects) and Group 2 (type 2 diabetic subjects).

**(A)**					
**Index**	***N***	**Mean**		**SD**	
PWVfinger	80	4.90 m/s		0.42 m/s	
SInew-RP	80	4.96 m/s		0.98 m/s	
SInew-DVP	80	4.38 m/s		0.95 m/s	
Total	240	4.75 m/s		0.86 m/s	

**Source of Variance**	**Sum of Squares**	**df**	**Mean Square**	**F-ratio**	***p*-value**
Between groups	16.455	2	8.227	12.112	<0.001
Within groups	160.987	237	0.679	-	-
Total	177.441	239	-	-	-
**(B)**					
**Multiple Comparisons**	***N***	**Mean**		**Mean Difference**	***p*-Value**
PWVfinger vs. SInew-RP	80 vs. 80	4.90 vs. 4.96 m/s		−0.058	0.658
PWVfinger vs. SInew-DVP	80 vs. 80	4.90 vs. 4.38 m/s		0.524 **	<0.001
SInew-RP vs. SInew-DVP	80 vs. 80	4.96 vs. 4.38 m/s		0.582 **	<0.001

** Significant at 0.001 level; *p* < 0.001. PWV_finger_—pulse wave velocity, measured by the PWV-ECG system [7]; SI_new_-RP—SI calculated using the new method based on decomposed radial pulse signals proposed in Equation (4); SI_new_-DVP—SI calculated using the new method based on decomposed DVP signals proposed in Equation (4).

**Table 4:** Correlations between SI_new_-RP/SI_new_-DVP and age, body weight, waist circumference, glycosylated hemoglobin, cholesterol, and triglyceride.

Parameter	SI_new_-RP	SI_new_-DVP
Age	r = 0.415 (*p* < 0.001)	r = 0.439 (*p* < 0.001)
Body weight	r = 0.257 (*p* = 0.041)	r = 0.233 (*p* = 0.038)
Waist circumference	r = 0.281 (*p* = 0.012)	r = 0.273 (*p* = 0.014)
Glycosylated hemoglobin	r = 0.400 (*p* < 0.001)	r = 0.365 (*p* = 0.001)
Cholesterol	r = 0.219 (*p* = 0.051)	r = 0.229 (*p* = 0.041)
Triglyceride	r = 0.333 (*p* = 0.003)	r = 0.307 (*p* = 0.006)

SI_new_-RP—SI calculated using the new method based on decomposed radial pulse signals proposed in Equation (4); SI_new_-DVP—SI calculated using the new method based on decomposed DVP signals proposed in Equation (4).

## Data Availability

All data structures are accessible upon demand from the authors.

## Supplementary Materials

The following supporting information can be downloaded at: https://www.mdpi.com/article/10.3390/jpm12111768/s1, Table S1: Comparison of stiffness indices (SIs) based on digital volume pulse from original pulse wave and IMF5 after EEMD [13,14]; Table S2: Stiffness index values based on pressure pulse among four different groups [15]; ΔT defined as in Figure 1 using only IMF5.
