# Supplementary material for: Calculation of an Improved Stiffness Index Using Decomposed Radial Pulse and Digital Volume Pulse Signals
Source: J Pers Med. 2022 Oct 26;12(11):1768. doi: 10.3390/jpm12111768 (PMC9694699; doi:10.3390/jpm12111768)
Supplement: Supplementary file 1 [file jpm-12-01768-s001.zip › jpm-1942244-supplementary.pdf]

Supplementary Materials

Table S1. Comparison of stiffness indices (SIs) based on digital volume pulse from original pulse wave and IMF5 after EEMD [13,14].

Healthy Subject	Age	Height (m)	△T (sec)	SI (m/sec)	△T with IMF5	SI with IMF5
A	24	1.73	0.235	7.36	0.205	8.44
B	52	1.70	0.180	9.44	0.231	7.36*
C	63	1.65	0.160	10.30	0.248	6.65*

* According to definition of arterial stiffness, the older subjects had higher SI values.

Table S2. Stiffness index values based on pressure pulse among four different groups [15]; ΔT defined as in Figure 1 using only IMF5.

Parameter	Younger subjects	Older subjects	Well-controlled diabetes	Poorly controlled diabetes
SI for IMF5, (m/sec)	4.83 ± 0.63	3.52 ± 0.66	3.22 ± 0.41#	2.77 ± 0.58#

According to definition of arterial stiffness, the subjects with T2DM should have significantly higher SI values.
